# Supplementary material for: Multimodal Deep Learning-Based Prognostication in Glioma Patients: A Systematic Review
Source: Cancers (Basel). 2023 Jan 16;15(2):545. doi: 10.3390/cancers15020545 (PMC9856816; doi:10.3390/cancers15020545)
Supplement: Supplementary file 1 [file cancers-15-00545-s001.zip › cancers-1975064-supplementary.pdf]

Supplemental Digital Content

Search strategy

PUBMED

("deep learning"[MeSH] OR "deep learning"[tiab] OR "deep-learning"[tiab] OR "deep neural network*"[tiab] OR "convolutional neural network*"[tiab] OR "recurrent neural network*"[tiab] OR "LSTM"[tiab] OR "long short-term memory"[tiab] OR "long short term memory"[tiab] OR "autoencoder"[tiab] OR "convnet"[tiab] OR "generative model"[tiab] OR "generative adversarial net*"[tiab] OR "diffusion model*"[tiab] OR "transformer"[tiab])
AND
("brain neoplasms"[MeSH] OR "central nervous system neoplasms"[MeSH] OR "brain neoplasm*"[tiab] OR "brain tumor*"[tiab] OR "brain tumour*"[tiab] OR "glioma*"[tiab] OR "astrocytoma*"[tiab] OR "ependymoma*"[tiab] OR "medulloblastoma*"[tiab] OR "primitive neuroectodermal tumor*"[tiab] OR "primitive neuroectodermal tumour*"[tiab] OR "craniopharyngioma*"[tiab] OR "meningioma*"[tiab])
AND
("diagnostic imaging"[MeSH] OR "radiograph*"[tiab] OR "xray*"[tiab] OR "x-ray*"[tiab] OR "computed tomogra*"[tiab] OR "mri"[tiab] OR "magnetic resonance*"[tiab] OR "ultraso*"[tiab] OR "positron emission tomogra*"[tiab])
AND
("predict*"[tiab] OR "prognos*"[tiab] OR “risk*”[tiab] OR "outcome*"[tiab] OR "complication*"[tiab])

EMBASE

("deep learning"/exp OR "deep learning":ti,ab OR "deep-learning":ti,ab OR "deep neural network*":ti,ab OR "convolutional neural network*":ti,ab OR "recurrent neural network*":ti,ab OR "LSTM":ti,ab OR "long short-term memory":ti,ab OR "long short term memory":ti,ab OR "autoencoder":ti,ab OR "convnet":ti,ab OR "generative model":ti,ab OR "generative adversarial net*":ti,ab OR "diffusion model*":ti,ab OR "transformer":ti,ab)
AND
("central nervous system cancer”/exp OR "brain neoplasm*":ti,ab OR "brain tumor*":ti,ab OR "brain tumour*":ti,ab OR "glioma*":ti,ab OR "astrocytoma*":ti,ab OR "ependymoma*":ti,ab OR "medulloblastoma*":ti,ab OR "primitive neuroectodermal tumor*":ti,ab OR "primitive neuroectodermal tumour*":ti,ab OR "craniopharyngioma*":ti,ab OR "meningioma*":ti,ab)
AND
("radiodiagnosis"/exp OR "radiograph*":ti,ab OR "xray*":ti,ab OR "x-ray*":ti,ab OR "computed tomogra*":ti,ab OR "mri":ti,ab OR "magnetic resonance*":ti,ab OR "ultraso*":ti,ab OR "positron emission tomogra*":ti,ab)
AND
("predict*":ti,ab OR "prognos*":ti,ab OR “risk*”:ti,ab OR "outcome*":ti,ab OR "complication*":ti,ab)

SCOPUS

(TITLE-ABS-KEY ("deep learning" OR "deep-learning" OR "deep neural network*" OR "convolutional neural network*" OR "recurrent neural network*" OR "LSTM" OR "long short-term memory" OR "long short term memory" OR "autoencoder" OR "convnet" OR "generative model" OR "generative adversarial net*" OR "diffusion model*" OR "transformer"))
AND
(TITLE-ABS-KEY (“central nervous system cancer” OR "brain neoplasm*" OR "brain tumor*" OR "brain tumour*" OR "glioma*" OR "astrocytoma*" OR "ependymoma*" OR "medulloblastoma*" OR "primitive neuroectodermal tumor*" OR "primitive neuroectodermal tumour*" OR "craniopharyngioma*" OR "meningioma*"))
AND
(TITLE-ABS-KEY ("radiograph*" OR "xray*" OR "x-ray*" OR "computed tomogra*" OR "mri" OR "magnetic resonance*" OR "ultraso*" OR "positron emission tomogra*"))
AND
(TITLE-ABS-KEY ("predict*" OR "prognos*" OR “risk*” OR "outcome*" OR "complication*"))
